# A Highly Sensitive Fluorescence and Screen-Printed Electrodes—Electrochemiluminescence Immunosensor for Ricin Detection Based on CdSe/ZnS QDs with Dual Signal

**DOI:** 10.3390/toxins14100710

**Published:** 2022-10-17

**Authors:** Shasha Feng, Wei Hu, Fubin Pei, Zhiwei Liu, Bin Du, Xihui Mu, Bing Liu, Qingli Hao, Wu Lei, Zhaoyang Tong

**Affiliations:** 1 State Key Laboratory of NBC Protection for Civilian, Beijing 102205, China; 2 School of Chemistry and Chemical Engineering, Nanjing University of Science and Technology, Nanjing 210094, China

**Keywords:** immunosensor, ricin, fluorescence, electrochemiluminescence, dual readout, CdSe/ZnS quantum dots

## Abstract

A sensitive dual-readout immunosensor for fluorescence and electrochemiluminescence (ECL) detection of ricin was established, which was combined with a streptavidin–biotin signal amplification system. CdSe/ZnS quantum dots with fine fluorescence and ECL properties were used as the dual-signal function probes of the sandwich immunocomplex. Under the optimum experimental conditions, the dual signal intensity increased significantly with the rise in ricin concentration. The fluorescence intensity of the senor exhibited a good liner relationship toward the ricin concentrations with 0.1~100 ng/mL and the limit of detection (LOD) was 81.7 pg/mL; taking ECL as the detection signal, the sensor showed a linear relationship with the ricin concentrations ranging from 0.01 ng/mL to 100 ng/mL and the LOD was 5.5 pg/mL. The constructed sensor with high sensitivity had been successfully applied to the detection of ricin in complex matrices with satisfactory recoveries. The proposed immunosensor model can be extended to the analysis and detection of others target proteins.

## 1. Introduction

Ricin is a highly toxic protein extracted from the seeds of the castor bean plant, and is also the only protein toxin prohibited by the international chemical weapons convention as well as the international biological and toxin weapons convention [1]. Because of its high toxicity, easy availability, and simple preparation, ricin is considered a high risk to public health and national security, making it a potential bioterrorist agent [2,3]. At present, there is no vaccine or inhibitor to block the toxic effect of ricin, and passive immunization is the only effective strategy to treat ricin poisoning [4]. Therefore, developing a rapid and sensitive method to detect ricin is very important for food safety protection and antiterrorism. At present, electrochemistry [5], fluorescence [6], electrochemiluminescence (ECL) [7], polymerase chain reaction [8], enzyme-linked immunosorbent assay (ELISA) [9], and surface plasmon resonance [10] have been used to detect ricin by immunoassay. However, these analysis methods only detect a single signal, which is easily affected by different sensing interfaces and environmental factors, resulting in false-negative or false-positive results [11]. The results of dual-signal detection can be mutually verified to effectively avoid false positives or false negatives, so as to provide more comprehensive and accurate results [12]. In traditional immunoassay methods, fluorescence and ECL strategies, are ideal methods for the detection of proteins, because they have the advantages of simple operation, rapid detection, and high sensitivity [13,14]. In addition, the ECL method also has the advantage of a low background signal [15]. ECL is the light emission caused by electrochemical reaction, which can avoid the interference of autofluorescence of biological samples in fluorescence immunoassay [12,16]. Moreover, the development of screen-printed electrodes has expanded the application range of ECL sensors and has broad application prospects in the development of miniaturized, integrated, and intelligent portable devices [17]. The sample volume required for screen printing electrodes is only tens of microliter. Fluorescence immunoassay does not require the use of electrodes and additional voltage, which can avoid the situation of unclean electrodes, unreasonable electrode preparation, and unstable applied voltage in the process of ECL operation [18]. Using fluorescence and ECL dual signals to analyze the target is conducive to mutual verification and improving the accuracy of the sensor. Among them, the label coupled with antigens or antibodies is responsible for signal conversion and will directly affect the sensitivity of the sensor. Thus, it is necessary to find photo/electric materials with dual functions.

Compared with traditional organic dyes, nanostructured materials have attracted extensive attention, especially quantum dots (QDs). QDs are one of the ideal candidates for optical labeled probes due to their remarkable features, including tunable fluorescence emissions, large specific surface area, narrow emission bandwidth, and wide excitation band [19,20]. In addition, QDs have become popular ECL emitters since the research on the electrochemical luminescence property of silicon QDs was published in 2002 [21]. It is reported that CdSe/ZnS QDs are the best fluorophores for biological applications, which are made of CdSe cores covered with ZnS [22,23]. The ZnS layer passivates the core surface, hinders its oxidation, prevents Cd or Se from entering the solution. It can also significantly improve the fluorescence quantum yield and chemical stability, and can cause a slight red-shift of the fluorescence emission [24,25,26]. In addition, CdSe/ZnS QDs possess a favorable ECL property with high quantum yields [27]. Moreover, functional groups on the surface of CdSe/ZnS QDs can be easily modified and be stably coupled with antibodies. As a result, CdSe/ZnS QDs are ideal candidates as dual-signal functional labels of immunosensors.

The complexity and diversity of samples determine the necessity to improve the efficiency of sample pretreatment procedures. Magnetic separation is one of the simplest and most effective methods for sample separation and enrichment [28]. Magnetic nanoparticles (MNPs) and magnetic microparticles (MMPs) have the advantages of uniform particle sizes, large specific surface areas, high magnetic separation efficiencies, and fast separation speeds, which are ideal carrier materials for sample separation and enrichment [29]. MNPs and MMPs have been widely used in many fields, such as biosensors [30], wastewater treatment [31], catalysis [32], and separation [33].

In order to further improve the sensitivity of the sensor, the streptavidin–biotin signal amplification system was introduced. The binding affinity (10^−14^ mol/L) between streptavidin and biotin exceeds that between antigens and antibodies [34,35]. One streptavidin has four specific binding sites with biotin, which can amplify the response signal and improve the sensitivity in immunoassay by labeling antigens, antibodies, or signal probes [35]. Therefore, many immunoassays utilize streptavidin-coated magnetic beads to immobilize biotinylated capture molecules for separation of immunocomplexes [36].

**Figure 1:**
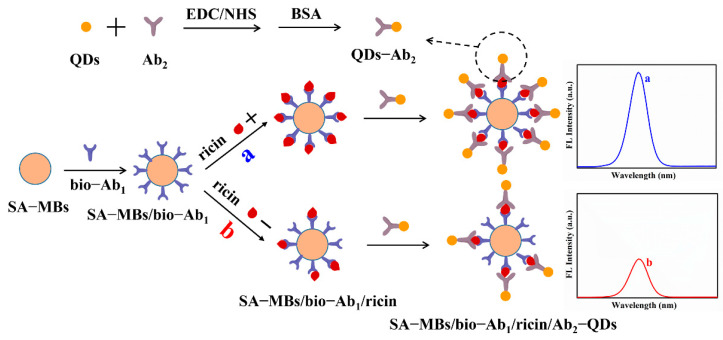
Schematic illustration of CdSe/ZnS QDs-based immunosensor combined with a streptavidin–biotin signal amplification system for ricin detection. The “+” and “−” represented the high and low concentration of ricin, respectively.

Herein, combined with a streptavidin–biotin signal amplification system, a fluorescence and ECL dual-signal immunosensor was proposed for detecting ricin. CdSe/ZnS QDs possessed good fluorescence and ECL properties and can be used as signal probes, laying the foundation for dual-signal detection. Streptavidin-modified Fe_3_O_4_ magnetic beads were used as carriers to load biotinylated capture antibodies for signal amplification and simplify the separation process. The sandwich conjugates (SA-MBs/bio-Ab_1_/ricin/Ab_2_-QDs) were formed when ricin appeared, which were tested on fluorescence and ECL platforms, respectively. The fluorescence and ECL signal gradually increased as the ricin concentration increased. To the best of our knowledge, there has been no report on dual-signal detection of ricin. The mechanism of the designed immunosensor is shown in Figure 1.

## 2. Results and Discussion

### 2.1. Characterization of QDs

**Figure 2:**
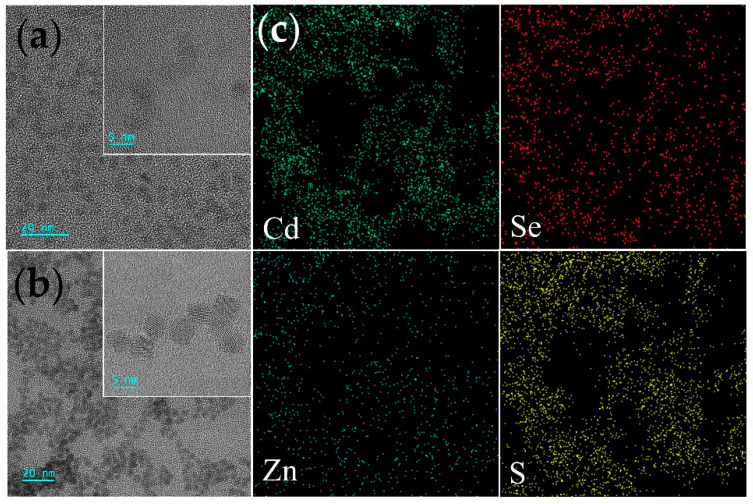
TEM images of CdSe QDs (**a**) and CdSe/ZnS QDs (**b**); elemental mapping images (**c**) of CdSe/ZnS QDs.

The transmission electron microscopic (TEM) images of CdSe QDs and CdSe/ZnS QDs, as well as elemental mapping images of CdSe/ZnS QDs, were displaced in Figure 2. It was obvious that CdSe QDs and CdSe/ZnS QDs were uniform spherical particles with good dispersion, and the particle sizes were about 4 nm and 5 nm, respectively. It can be seen from the high-resolution TEM (HRTEM) images that the QDs had clear lattice planes, confirming the good crystallinity of the materials. Additionally, the lattice of CdSe/ZnS QDs was directly extended without an interface, which was consistent with the coherent epitaxial growth mechanism [37]. The existence of CdSe/ZnS QDs was demonstrated by the elemental mapping pictures (Figure 2c) of Cd, Se, Zn, and S elements with clear color contrast.

**Figure 3:**
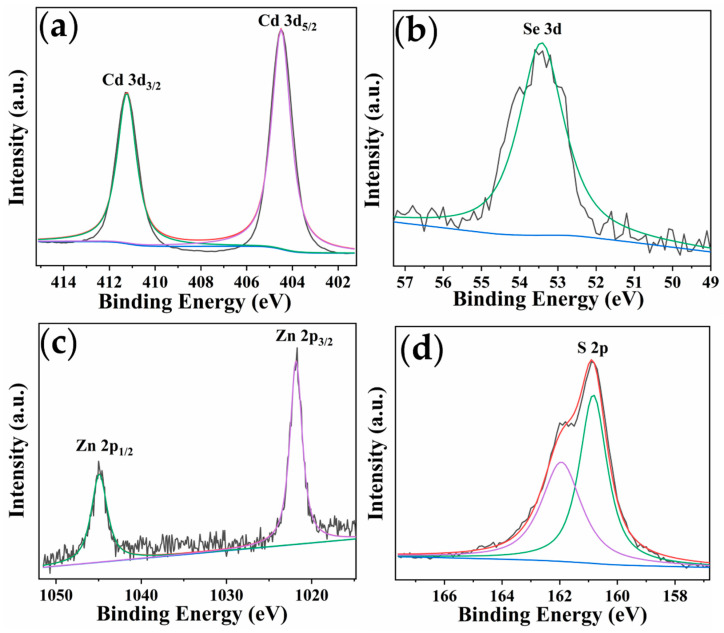
High resolution XPS spectra of Cd 3*d* (**a**), Se 3*d* (**b**), Zn 2*p* (**c**), and S 2*p* (**d**).

The X-ray photoelectron spectroscopy (XPS) measurements were performed to analyze the composition and chemical state of the QD surface (Figure 3). The peaks at 411.24 eV and 404.49 eV were attributed to Cd 3*d_3/2_* and Cd 3*d_5/2_*, respectively. The binding positions of Se 3*d* were located at around 53.43 eV. The Zn 2*p* doublet were observed at binding energies of 1044.92 eV and 1021.83 eV, corresponding to Zn 2*p_1/2_* and Zn 2*p_3/2_* orbitals of Zn^2+^. The asymmetric S 2*p* spectrum was fitted to two peaks, at 161.94 eV and 160.83 eV, which were assigned to S 2*p_3/2_* and S 2*p_1/2_*, respectively [38]. The results confirmed that CdSe/ZnS QDs were prepared successfully.

### 2.2. Optical Properties of CdSe/ZnS QDs

**Figure 4:**
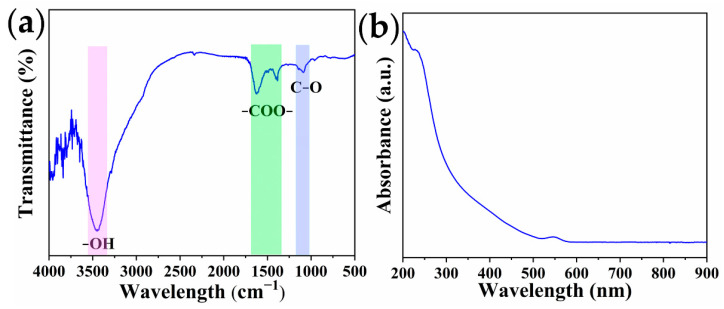
FTIR (**a**) and UV-vis absorption spectrum (**b**) of CdSe/ZnS QDs.

Fourier transform infrared spectra (FT-IR) was used to confirm functional groups on the surface of the QDs. As shown in Figure 4a, the characteristic peak observed at 3443 cm^−1^ was assigned to the -OH stretching vibration. The asymmetric and symmetric stretching of -COO- were located at 1621 cm^−1^ and 1386 cm^−1^, respectively. The peak at 1085 cm^−1^ was related to the stretching vibration of C-O. No characteristic peak of -S-H stretching was observed at 2550 cm^−1^, which indicated that 3-mercaptopropionic acid (MPA) molecules were successfully capped onto the surface of the QDs through S-Cd bonds. The above results indicated that a carboxyl group was introduced by coating MPA on to the QD’s surface, which created the conditions for attaching antibodies on the surface of the QDs. As illustrated in Figure 4b, the prepared QDs exhibited two characteristic absorption peaks, at 232 nm and 546 nm.

**Figure 5:**
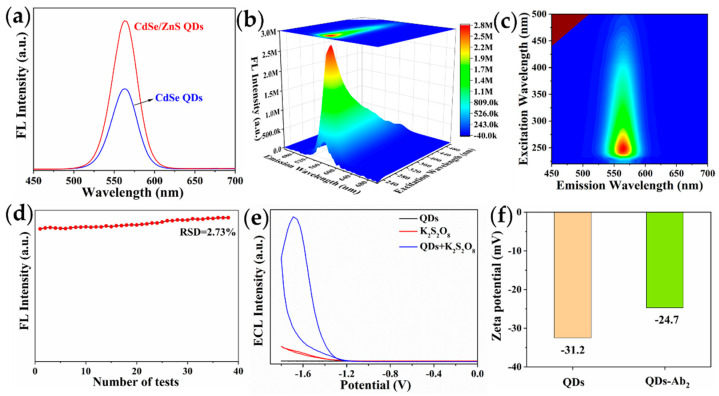
(**a**) FL emission spectra of CdSe QDs and CdSe/ZnS QDs; (**b**) three-dimensional fluorescence spectra, (**c**) fluorescence contour spectra, and (**d**) stability of CdSe/ZnS QDs; (**e**) ECL spectra under different conditions; (**f**) zeta potential of QDs and QDs-Ab_2_.

The fluorescence and ECL performances of CdSe/ZnS QDs were investigated. As can be seen from Figure 5a, the fluorescence signal intensity of CdSe/ZnS QDs was almost twice that of CdSe QDs. This was attributed to the formation of ZnS passivation layer on the surface of CdSe QDs, thus eliminating the trap state on the surface of the QDs [39]. The emission wavelengths of CdSe QDs and CdSe/ZnS QDs were 562 nm and 564 nm, with the same full width at half maximum (FWHM) of 37 nm, respectively, due to the red-shift of the emission wavelength of the QDs with increasing particle size. The three-dimensional fluorescence spectra (Figure 5b,c) of the CdSe/ZnS QDs showed that the optimal excitation wavelength was 251 nm, and had obvious excitation independence characteristics. Moreover, the QDs were tested 38 times, continuously, and the relative standard deviation (RSD) was 2.37% (Figure 5d), indicating that the QDs had good stability and resistance to photobleaching. As displayed in Figure 5e, the ECL signal of individual QDs cannot be observed. In addition, the 0.05 mol/L K_2_S_2_O_8_ solution produced a very weak peak. When K_2_S_2_O_8_ and QDs existed simultaneously, a strong ECL signal appeared, indicating that the ECL signal of the QDs belonged to the cooperative reaction type (coreactant luminescence mechanism). The luminescence mechanism of QDs may be as follows [40]:

(1) $$\mathrm{QDs}+{\mathrm{ne}}^{-}\to {\mathrm{nQDs}}^{-}$$

(2) $$S_{2}O_{8}^{2-}+e^{-}\to {\mathrm{SO}}_{4}^{2-}+{\mathrm{SO}}_{4}^{-}$$

(3) $${\mathrm{QDs}}^{-}+{\mathrm{SO}}_{4}^{-}\to {\mathrm{QDs}}^{*}+{\mathrm{SO}}_{4}^{2-}\;$$

(4) $${\mathrm{QDs}}^{*}\to \mathrm{QDs}+hv\;$$

### 2.3. Zeta Potential

Zeta potential of the QDs and QDs-Ab_2_ were recorded to verify the successful preparation of the QDs-Ab_2_ conjugates. As demonstrated in Figure 5f, the zeta potential of the QDs was −31.2 mV, which was due to the large number of carboxyl groups on the surface of the QDs. After the Ab_2_ was coupled with the QDs, the zeta potential changed from −31.2 mV to −24.7 mV, indicating that the QDs-Ab_2_ conjugates were successfully prepared [41].

### 2.4. Optimization of Experimental Conditions

#### 2.4.1. Optimization of Reaction Time of ZnS

**Figure 6:**
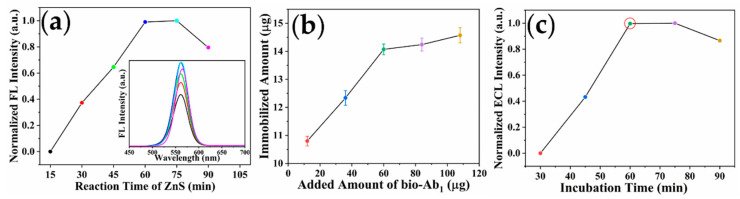
(**a**) Effect of reaction time of ZnS on fluorescence intensity of CdSe/ZnS QDs; (**b**) optimization of added amount of bio-Ab_1_; and (**c**) incubation time between QDs-Ab_2_ conjugates and ricin.

The performance of the sensor is critically dependent on the optical properties of fluorophores. Figure 6a showed that the fluorescence signal increased as the reaction time of ZnS lengthened. When the reaction time of ZnS exceeded 60 min, the fluorescence intensity trended downward. Because the ZnS shell thickness increased with the increase in reaction time, the defects on the surface of quantum dots can be effectively reduced, which can enhance their fluorescence performance. However, new defects will appear on the surface of the QDs with excessive growth of the shell thickness, resulting in the decrease in their fluorescence performance [25]. Therefore, 60 min was selected as the reaction time of ZnS.

#### 2.4.2. Optimization of Added Amount of bio-Ab_1_

As a capture antibody, the immobilized amount of bio-Ab_1_ on streptavidin-coated magnetic beads (SA-MBs) will directly affect the number of captured antigens, thus affecting the sensitivity of the sensor. The immobilized amount of Ab_1_ was calculated according to the absorbance of the Ab_1_ solution before and after incubation with SA-MBs [42].

(5) $$R_{Binding\;Ratio}=\frac{A280_{before}-A280_{after}}{A280_{before}}\times 100\%$$

(6) $$\mathrm{Immobilized}\;\mathrm{Amount}\;\left(µg\right)=\mathrm{Added}\;\mathrm{Amount}\;\left(µg\right)\times R_{Binding\;Ratio}$$

As illustrated in Figure 6b, with the increase in bio-Ab_1_ added amount (12, 36, 60, 84, and 108 µg), the amount of antibodies immobilized on the surface of the SA-MBs gradually increased. When the amount of bio-Ab_1_ added reached 60 µg, the amount of bio-Ab_1_ immobilized on the surface of SA-MBs reached saturation. Thus, the optimal added amount of bio-Ab_1_ was 60 µg.

#### 2.4.3. Optimization of Incubation Time between QDs-Ab_2_ Conjugates and Ricin

The incubation time between the QDs-Ab_2_ conjugates and ricin will affect the number of the bound labeled probes on the ricin, thus affecting the performance of the sensor. The immunosensors under different conditions were used to detect 100 ng/mL ricin. As shown in Figure 6c, the fluorescence signal increased with the increase in incubation time (30, 45, 60, 75, and 90 min). After incubation for 60 min, the fluorescence signal was almost unchanged, indicating that the binding between ricin and QDs-Ab_2_ conjugates had reached equilibrium. Therefore 60 min was selected as the optimal incubation time between the QDs-Ab_2_ conjugates and ricin.

### 2.5. Analytical Performance of the Immunosensor

**Figure 7:**
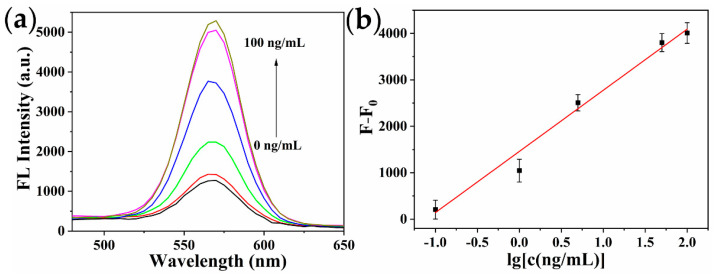
(**a**) Fluorescence spectra of the immunosensor in the presence of various concentrations of ricin. Ricin concentration: 0, 0.1, 1, 5, 50, and 100 ng/mL. (**b**) The calibration curve between fluorescence intensity and ricin concentration. Error bars were based on the results of three parallel experiments.

**Figure 8:**
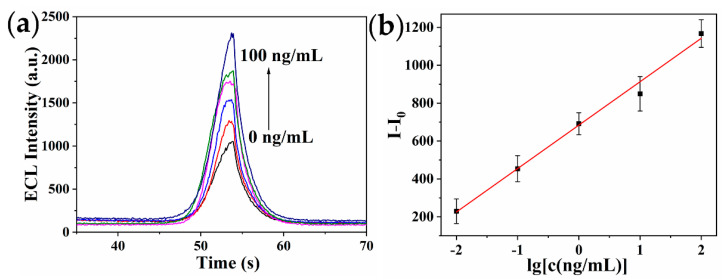
(**a**) ECL response curves of the immunosensor at various concentrations of ricin. Ricin concentration: 0, 0.01, 0.1, 1, 10, and 100 ng/mL ricin. (**b**) The calibration curve between ECL intensity and ricin concentration.

Under the optimized conditions, the performance of the fluorescence and ECL dual-signal immunosensor was investigated for detecting various concentrations of ricin. As represented in Figure 7 and Figure 8, the detection signal increased as ricin concentration increased. The prepared immunosensor had a good linear relationship between fluorescence intensity and ricin concentration in the range of 0.1~100 ng/mL, and the linear regression equation was F − F_0_ = 1316.59·lg[c(ng/mL)] + 1458.47 (R^2^ = 0.990). The limit of detection (LOD) was 81.7 pg/mL, which was calculated based on the average response of the negative control (n = 11) plus three times the standard deviation [43]. The ECL intensity was proportional to the logarithmic value of ricin concentration ranging from 0.01~100 ng/mL, with the linear regression equation of I − I_0_ = 229.26·lg[c(ng/mL)] + 683.91 (R^2^ = 0.994). The LOD was 5.5 pg/mL. Compared with the fluorescence method, the ECL method had higher sensitivity, wider linear range, and required less solution volume and more portable instruments, making it more suitable for outdoor analysis and detection.

### 2.6. Selectivity, Stability, and Reproducibility of the Immunosensor

**Figure 9:**
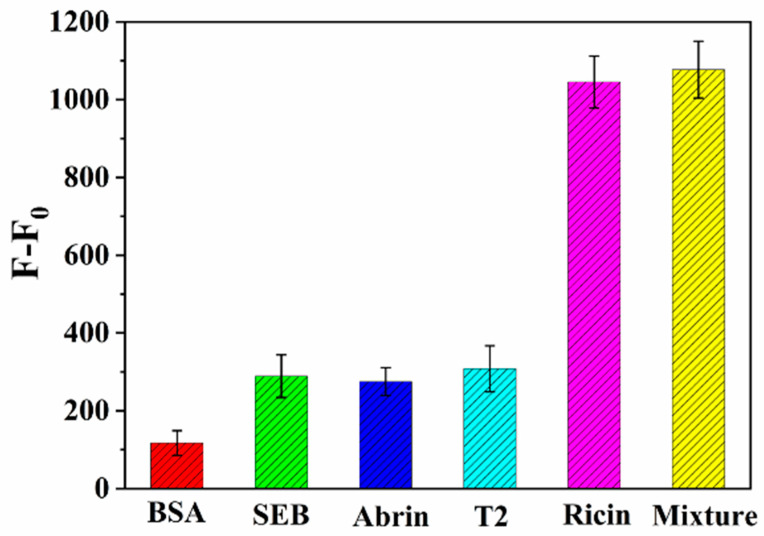
The selectivity study of the immunosensor.

To investigate the specificity of the immunosensor, 10 ng/mL bovine serum albumin (BSA), staphylococcal Enterotoxins B (SEB), T2-toxin (T2), and 1 ng/mL abrin were selected as interferences. As shown in Figure 9, the signal of interferences was much smaller than that of ricin, and the influence can almost be ignored. At the same time, it can be seen that the fluorescence signal of the mixture solution of above each substance with 1 ng/mL ricin was basically consistent with the signal of 1 ng/mL ricin detected. The result indicated that the immunosensor had good specificity.

The reproducibility of the immunosensor was evaluated by five parallel electrodes prepared in the same way for fluorescence analysis of 5 ng/mL ricin. The RSD of five results was less than 5%, demonstrating that the immunosensor had good reproducibility.

### 2.7. Detection of Ricin in Simulated Samples

In order to study the practicability of the proposed immunosensor in complicated samples, river water, soil, and tap water samples were used as simulated samples for detection by the immunosensor. The recovery of samples was determined by the standard addition recovery method. As shown in Table 1, the recovery was between 91.6% and 109.6%. These satisfactory experimental results indicated that the sensor was reliable in practical application.

## 3. Conclusions

In summary, a new fluorescence and ECL dual-signal immunosensor for ricin detection was established, combined with a streptavidin–biotin signal amplification system. CdSe/ZnS QDs were used as dual-signal probes to transmit fluorescence and ECL signals. The proposed immunosensor demonstrated good selectivity, high sensitivity, and low LOD. The dual-signal immunosensor can improve the reliability of the detection results. The immunosensor had been successfully used to detect ricin in complicated samples, which was expected to expand the application prospects.

## 4. Materials and Methods

### 4.1. Materials and Apparatus

Dynabeads^TM^ M-280 Streptavidin (10 mg/mL, SA-MBs) was obtained from Thermofisher Scientific Co., Ltd. (Waltham, MA, USA). Cadmium chloride hydrate (CdCl_2_·2.5H_2_O), triethanolamine (TEA), MPA, zinc acetate (Zn(CH_3_COO)_2_), thiourea, potassium persulfate (K_2_S_2_O_8_), and 2-morpholinoethanesulfonic acid (MES) were provided by Aladdin Regents Co., Ltd. (Shanghai, China). Ricin, SEB, T2, and abrin were provided by Beijing Hapten and Protein Biomedical Institute (Beijing, China). Anti-ricin polyclonal antibodies/bio (bio-Ab_1_) were prepared in our lab. Anti-ricin monoclonal antibodies (2R1, detection antibodies, Ab_2_) were purchased from HyTest Ltd. (Turku, Finland). Bovine serum albumin (BSA) and phosphate buffer solution (PBS, 0.01 mol/L, pH = 7.2) were the products of Beijing Solarbio Science & Technology Co., Ltd. (Beijing, China). N-hydroxysulfosuccinimide (NHS), 1-ethyl-3-(3-dimethylaminopro-pyl) carbodiimide hydrochloride (EDC), and sodium selenite (Na_2_SeO_3_) were obtained from Sigma-Aldrich Chem. Co. (Hamburg, Germany). All reagents used in this experiment are of analytical purity and can be used directly without further purification. Ultrapure water (18.2 MΩ/cm) was prepared using a arium 611 ultrapure water system (Sartorius, Goettingen, Germany). 

Fluorescence spectra of QDs were recorded by FLS1000 spectrophotometer (Edinburgh, UK). The fluorescence spectra of the sensor were measured on a Spark Multi-Mode Microplate Reader (Tecan, Austria). FT-IR were tested by a Nicolet iS50 (Thermo Scientific, Waltham, MA, USA). UV–vis absorption spectrum was recorded by Biomate 3S UV-Visible Spectrophotometer (Thermo Scientific, Waltham, MA, USA). TEM images were obtained with a JEM-F200 (JEOL, Akishima, Japan). XPS survey spectra were performed with Thermo Scientific K-Alpha (Waltham, MA, USA). HS-3 vertical mixer was obtained by Ningbo Scientz Biotechnology Co., Ltd. (Ningbo, China). The TE100 screen-printed carbon electrodes (SPCEs) were purchased from Zensor Research and Development Co., Ltd. (Taiwan, China). The 96-well, black, flat-bottom polystyrene high-bind microplates were purchased from Corning Incorporated (Corning, NY, USA). The ECL measurement was carried out with an MPI-ECL analyzer from Xi’an Remex Analysis Instruments Co., Ltd. (Xi’an, China).

### 4.2. Preparation of the Soil Sample

A soil sample was collected near the laboratory, after which it was air-dried, ground, and then dispersed in water for ultrasonic for 30 min. The sample was centrifuged to remove sand and impurities, and the supernatant was properly diluted before testing.

### 4.3. Synthesis of CdSe/ZnS QDs

CdSe QDs were synthesized by using cadmium chloride and Na_2_SeO_3_ as precursors and MPA as capping agent molecules (refluxing for 24 h) [44]. Then, 4.3 mL ZnS shell stock solution containing 0.016 mol/L Zn(CH_3_COO)_2_ and 0.016 mol/L thiourea was added to CdSe QDs and refluxed at 100 °C for 60 min to obtain CdSe/ZnS QDs. Then, CdSe/ZnS QDs were centrifuged and concentrated in an ultrafiltration tube (10 kD) and then dissolved to half of the original volume with water and stored at 4 °C for standby.

### 4.4. Preparation of SA-MBs/bio-Ab_1_

For the preparation of SA-MBs/bio-Ab_1_, 200 µL SA-MBs (10 mg/mL) were washed and magnetically separated three times with 0.01 mol/L PBS before being dispersed in 500 µL 0.01 mol/L PBS (pH = 7.4) containing 0.12 mg/mL bio-Ab_1_. After rotating at room temperature for 30 min, the mixture was washed three times with 0.01 mol/L PBS (pH = 7.4) to remove the excess unbounded bio-Ab_1_ by magnetic separation. The prepared SA-MBs/bio-Ab_1_ was redispersed in 2 mL 0.01 mol/L PBS (pH = 7.4) and stored at 4 °C for further use.

### 4.5. Preparation of QDs-Ab_2_ Bioconjugates

CdSe/ZnS QDs and Ab_2_ were coupled by EDC/NHS. Typically, 200 µL CdSe/ZnS QDs were added to 1 mL 0.1 mol/L MES buffer solution containing 600 µL EDC (10 mg/mL) and 400 µL NHS (10 mg/mL), and activated under ultrasonic treatment for 30 min. Then, the mixed solution was centrifuged and washed twice with 0.01 mol/L PBS (pH = 7.4) to remove unreacted EDC and NHS. The activated QDs were redispersed in 1.0 mL 100 µg/mL Ab_2_, rotated at room temperature for 4 h, centrifuged, and washed twice with 0.01 mol/L PBS (pH = 7.4) containing 0.05% BSA to remove the excess antibodies. The QDs-Ab_2_ bioconjugates were redispersed in 1.5 mL 0.01 mol/L PBS (pH = 7.4) containing 0.05% BSA to block the nonspecific recognition sites, which were stored at 4 °C (avoiding light in the whole process).

### 4.6. Detection of Ricin

The prepared SA-MBs/bio-Ab_1_ (100 µL) was mixed with 200 µL ricin of different concentrations and rotated at 37 °C for 30 min. Then, the SA-MBs/bio-Ab_1_/ricin conjugates were washed three times with 0.01 mol/L PBS (pH = 7.4) by magnetic separation. Then, 200 µL of CdSe/ZnS-Ab_2_ bioconjugate dispersion was added and rotated for 1 h at room temperature, which were washed four times with 0.01 mol/L PBS (pH = 7.4) by magnetic separation. The SA-MBs/bio-Ab_1_/ricin/Ab_2_-QDs conjugate dispersion was redispersed in 120 µL 0.01 mol/L PBS (pH = 7.4) for fluorescence test. The above process was repeated, and the SA-MBs/bio-Ab_1_/ricin/Ab_2_-QDs conjugate dispersion was redispersed in 50 µL 0.01 mol/L PBS (pH = 7.4) containing 0.05 mol/L K_2_S_2_O_8_ for ECL measurement.

### 4.7. Fluorescence and ECL Measurements

The sensitivity of the sensor was determined by the enzyme-labeled instrument and the ECL analyzer (in addition to the sensitivity test of the sensor, other performances only recorded the fluorescence signal). Parameter setting of enzyme-labeled instrument: using the fluorescence intensity scanning mode, the type of enzyme-labeled plate was costar 96 black, the emission wavelength range was 480~650 nm, the step was 5 nm, and the gain value was 100. Then, 100 μL SA-MBs/bio-Ab_1_/ricin/Ab_2_-QDs conjugate dispersion was added to the enzyme-labeled plate. The fluorescence spectra were recorded in 0.01 mol/L PBS (pH = 7.4) with an excitation wavelength of 360 nm. For ECL, 20 μL SA-MBs/bio-Ab_1_/ricin/Ab_2_-QDs conjugate dispersion was evenly coated on the working surface of the SPCE. The ECL measurements were carried out by cyclic voltammetry (CV) from 0 V to –1.8 V with a scan rate of 100 mV/s in 0.01 mol/L PBS (pH = 7.4) containing 0.05 mol/L K_2_S_2_O_8_, and the photomultiplier tube was fixed at 700 V.

## Figures and Tables

**Table 1:** Recovery tests for ricin in simulated samples (n = 3).

Sample	Original (ng/mL)	Added (ng/mL)	Found (ng/mL)	Recovery %	RSD %
River water	0	1	0.916	91.6	3.58
Soil	0	1	1.096	109.6	4.37
Tap water	0	1	1.042	104.2	3.29

## Data Availability

Not applicable.
